# Perception of inpatients following remission of a manic episode in bipolar I disorder on a group-based Psychoeducation program: a qualitative study

**DOI:** 10.1186/s12888-018-1614-1

**Published:** 2018-01-30

**Authors:** Runsen Chen, Yingjun Xi, Xue Wang, Yaqiong Li, Yuyang He, Jiong Luo

**Affiliations:** 1 The National Clinical Research Center for Mental Disorders & Beijing key Laboratory of Mental Disorders, Beijing Anding Hospital, Capital Medical University, No. 5 Ankang Lane, Dewai Avenue, Xicheng District, Beijing, 100088 China; 2 Department of Psychiatry, University of Oxford, Oxford, UK

**Keywords:** Bipolar disorder, Psychiatric inpatients, Group-psychoeducation, Qualitative, Cultural adaptations

## Abstract

**Background:**

This forms the first study of a group-based psychoeducation program for inpatients following remission of a manic episode in patients suffering from bipolar I disorder in a Chinese population. The aim was to explore the patient’s perspectives of the program and their suggestions regarding ways to improve the intervention in the future.

**Methods:**

Semi-structured and in-depth interviews were conducted with 15 participants who had participated in 8 sessions of a group psychoeducation program over 2 weeks. The verbatim transcripts of those interviews were analysed using thematic analysis.

**Results:**

Five themes emerged from the data, including the patients’ perception of participating in the program, their perception of the setting, perception of participating in a group program, perception of the learning content and of the outcome of participating in the program.

**Conclusions:**

The results presented here describe how the short-term group psychoeducation program was experienced by the patients. Recommendations are also offered to improve the setting, content, and delivery. Our findings provide evidence that the program is beneficial for manic patients with bipolar I disorder, and this intervention warrants further research especially in a Chinese population. If these benefits are confirmed in future studies, this program could be incorporated into routine psychiatric inpatient care in China.

## Background

Bipolar disorder type I (BD-I) is the sixth leading cause of disability globally [1]. Manic episodes are a common feature of BD-I and the lifetime prevalence is about 1.5% in European countries [2]. The lifetime prevalence of BD-I in China is about 0.09% in the year of 2017 [3]. Acute manic episodes usually require emergency admission to a psychiatric hospital to facilitate rapid recovery [4]. A range of quantitative researches indicate that group-based psychoeducation interventions guide patients to greater awareness of relapse features [5], reduce time spent in bipolar episodes [6], raise treatment adherence levels and improve quality of life [7]. Poole, Smith and Simpson [8] conducted a qualitative study on the perspectives of bipolar outpatients’ enrolled in a group-based psychoeducation program in the UK, and they found that psychoeducation had a positive impact on patients’ medication adherence, social support, knowledge and acceptance of BD and access to the services. However, there are few psychoeducational studies have been conducted in psychiatric inpatient settings, inpatients are more severely impaired than outpatients [9] and most patients suffering from manic episodes require hospital admission. It is unclear whether psychoeducational programs may benefit psychiatric routine care in severely manic patients in the literature. Dropout rates are very high in the outpatient settings, perhaps due to the long duration of psychoeducation programs [10], hence the present study adjusted the length of treatment to examine whether a short duration psychoeducational program could reduce drop-out rates whilst resulting in similar benefits.

Furthermore, research indicates a need to examine psychoeducation for BD as a tool to engage people from diverse ethnic backgrounds [8]. Chinese populations hold particularly stigmatized attitudes towards mental illness because of Confucian beliefs [11, 12]. The diagnosis and treatment of mental disorder leads to marginalization due to perceived humiliation within the community [13]. Thus, many discharged patients no longer feel the need to see a doctor regularly and go off medication use as a consequence. Psychoeducation may be an important way to address the stigma and other barriers to mental health treatment amongst those inpatients [14]. There is also evidence from a systematic review suggesting that mental health interventions targeted to a specific cultural group are four times more effective than interventions that do not target such cultural differences. The same is true for interventions conducted in the patients’ native language (if other than English), being twice as effective than those using English [15]. It is unclear whether culturally adapted group-based psychoeducational programs are acceptable and feasible for Chinese patients.

As the present study is the first to conduct a group-based psychoeducation program for BD-I inpatients in China, a qualitative evaluation is the most appropriate way to understand unique cultural elements of engaging with the intervention [16]. Therefore, we aimed to explore the experiences of Chinese patients with bipolar disorder of being in a culturally adapted short group-based psychoeducation program and their perceptions of elements such as content, teaching, delivery methods, impact and suggestions for improvement of the program in China.

## Method

### Group-based intervention

The program was delivered by the Beijing Anding Hospital affiliated Capital Medical University research team. All BD-I patients admitted to the inpatient unit and who received standard hospitalized treatment between September 2015 and April 2016 were approached for participation. Participants were referred to the program when their clinical symptoms were alleviated [as defined by a Young Mania Rating Scale (YMRS) score of less than 8] and during their discharge period. The program delivered eight sessions of 40–60 min duration in two weeks. Three psychiatrists and two clinical psychologists directed these sessions and the facilitators were trained by an academic expert in psychoeducation. The psychoeducation handbook was culturally adapted from Colom and Vieta [17], and the group sessions included the following topics: Session 1: introduction of BD disease knowledge, such as biological etiology, epidemiology and concepts; Session 2: definition of mania and hypomania, depression mixed state and psychotic symptoms; Session 3: biological rhythm and manic/depressive episode; Session 4: the role of pharmacological treatment and different types of medication; Session 5: medication adherence and monitoring, electroconvulsive therapy and psychotherapy; Session 6: stress management, problem coping strategies and interpersonal relationships; Session 7: recurrent signal, early detection of episodes and how to seek help; Session 8: review and assessment, how to establish a management plan and how to monitor daily mood. *The psychoeducation materials in Chinese are available for request by email from the corresponding author.*

### Sample

Purposive sampling was used to select 13 participants who successfully completed the program and 2 participants who did not complete the program. Participants who had been discharged from the hospital were invited to take part in this interview process through a letter/email, which was sent along with the informed consent form. If the participants were willing to take part in the study, they had two weeks to contact the author by telephone and arrange a suitable time for the interview in the hospital. Resident participants were recruited from the inpatients ward directly after they completed the program. Data saturation was achieved at the 12nd participant. Three more interviews were conducted to examine the saturation [18], giving a total sample of 15 participants (Table 1). Each individual interview lasted 40 to 60 min and was conducted by the authors. All interviews were audio-recorded.

**Table 1:** Socio-demographic characteristics of participants

Patients characteristics *N* = 15	
Gender	
Male	4
Female	11
Age	
Range	18–51
Mean	34
Education	
Middle school	3
High school	4
College and above	8
Times of Episode	
Mean	3.7

### Data collection

This study aimed to understand underlying themes in patients’ perspectives of the group-based psychoeducation program and any impact of the intervention. Hence, researchers used semi-structured interviews to obtain the data and used thematic analysis as this was the most appropriate method for qualitative analysis [19]. All interviews were conducted at the affective disorder inpatient unit. The interview questions were developed through literature review and discussion with qualitative experts. The expert panels include two experts in qualitative research and psychoeducation. The interview questions were revised after their review. Also, in order to compare the perception of Chinese patients with those of European patients, some of the questions were adapted from Poole, Smith and Simpson [8]. Some sample questions discussed in the interviews were as follows: How did you feel while participating in this program (initial question)? Would you recommend the program to others? How do you feel about the learning environment? How do you feel about the facilitators? What do you think of the learning content? How can this content be improved further? What is your experience of the group? What has been the benefit (s) of participating in the program?

### Analysis

The transcripts were analyzed according to the thematic analysis method described by Braun and Clarke [19]. There are six phases of thematic analysis:

- familiarizing with data;
- generating the initial codes;
- searching themes from text;
- reviewing themes;
- defining and naming the themes;
- producing the report.

To ensure that conformability was established, two independent authors separately reviewed the coding and themes, one author with a background in clinical psychology, and a second author with a background in psychiatry [19]. Related words or sentences representing an underlying idea were identified and coded into a category for the analysis of emerging sub-themes [20]. Also, these two authors held several meetings to compare their notes on themes and sub-themes in order to achieve consensus upon an accurate interpretation of the data. The credibility of the data was established through a check by another staff member.

### Ethics

This study was approved by the Human Research and Ethics Committee of Beijing Anding Hospital, Capital Medical University. Informed consent, including permission to record the interviews, was obtained from the participants. The participants were also informed that they could withdraw from the study at any time without reason or penalty.

## Results

Five themes emerged from the thematic analysis: (1) perception of participating in the program; (2) perception of the setting; (3) perception of being in a group; (4) perception of learning content; and (5) perception of the outcome of participating in program.

### Theme 1: Perception of participating in the program

#### Sub-theme 1: Engagement

For those participants who were willing to complete the program, the most common reason for doing so was their wish to understand more about their illness.

“I have a better understanding of the illness, why not? It’s cost-free” (Participant 10).

#### Sub-theme 2: Recommending the program to others

All participants would recommend the program to others. They commented that people would experience a range of benefits if they participated in this program.

“I feel so lucky that I was selected to attend this program, I would definitely recommend the program to others I know suffering from a bipolar disorder.” (Participant 6)

#### Sub-theme 3: Suggestions for the program

Some participants suggested that the research team should have made a comprehensive booklet for patients who wanted to learn further in their free time.

“Can you print the handout for me before I am discharged from the hospital? I need it. I can review it when I am at home.” (Participant 6)

Furthermore, participants expressed interest in any follow-up studies, where they hoped to learn suggestions for dealing with their illness.

“I would like to have access to more up-to-date information about my illness and treatments from the doctor.” (Participant 11)

### Theme 2: Perceptions of the setting

#### Sub-theme 1: Learning environment

Most participants reported that the classroom was relaxed, and that the light background music created a pleasant environment.

“This ambiance with light music enhanced my mood and allowed me to focus on the class.” (Participant 7)

“A big table can fit all of the people (group members) and I felt it’s a teamwork and our heart tights junctions.” (Participant 10)

Some participants suggested ways in which the setting could be improved, such as the need to create a quieter environment to avoid potential distractions and the need to improve lighting conditions to improve the readability of the powerpoint slides.

**“**um….the light was too dim, I felt sleepy and a bit depressed....it was hard to concentrate on the class. What I suggested is a change to a brighter light.” (Participant 2)

“You know, I would wonder sometimes if I was hearing the noise from outside. I hope it’s quiet.” (Participant 3)

Also, some participates suggested that they required a short break, perhaps with a light refreshment, in order to increase their attention and participation.

“The session was long, and we should have had five minutes to rest in between.” (Participant 1)

#### Sub-theme 2: Facilitators

Most of the participants reported that the facilitators performed at an expert level and their speech was clear and well structured. Participants observed that the facilitator were friendly, responsible, and were attentive and respectful listeners.

“The class was very organized; the devices, such as the laptop, projector and practice booklet, were well arranged by those doctors and every time they came to pick us up from the ward to the classroom, they were always on time; I appreciated that.” (Participant 8)

However, the suggestions included a range of recommendations. The facilitator’s slides should have included more interesting, flashy information and photos. Indeed, text-focused presentations may create the impression that the presentation is ‘dry’ or overly technical and boring.

“There are too many texts in some slides, I found the class quite boring.” (Participant 9)

Furthermore, the facilitator should have allowed more time to answer questions from the class.

“I had many questions on the medication section; I wanted to ask more questions to the teacher but the time was ending.” (Participant 9)

Also, it was suggested that the facilitators should improve their class-management skills, as participants reported discouragement or distress due to others’ negative or irrelevant comments.

“Sometimes the class was in a little bit of chaos and I felt irritable towards the group members who were speaking loudly and dominating the group.” (Participant 1)

Finally, it was suggested that facilitators could start each lesson with an offer to briefly review prior contents, as this could have enhanced understanding and retention.

“However, some contents are easy to forget, I wish the facilitator had repeated things again at the beginning of the class.” (Participant 8)

#### Sub-theme 3: Time

Participants preferred to attend the class in the afternoon rather than in the morning, due to sleepiness.

“You know, we have to wake up at 6 am during the routine hospitalization. In the following 3 hours, I have to eat breakfast, read some books and play table tennis. Whenever the class started at 10 am, I felt tired and sleepy. So, I prefer to attend the 3pm class in the afternoon as I felt more energized then after my 2pm nap.” (Participant 5)

Most participants agreed that each session should take about 40 min.

“Sometimes the class took longer than 40 minutes and I felt restless.” (Participant 6)

Sessions had intervals of 2 to 3 days; because if the gap between the sessions is too long, some participants may lose interest and patience in the program.

### Theme 3: Perception of participating in a group

#### Sub-theme 1: Group-versus individual-based intervention

Most of the participants commented that the group-based psychoeducation was favorable to the individual psychoeducation intervention. They reported that group-based programs can help participants realise that they are not alone in suffering from BD.

“I am not only the one who has bipolar disorder; many people around me have the same problems. Within the group, we can discuss bipolar disorder.” (Participant 8)

They reported benefits of exposure to a wider range of perspectives on their situation when hearing from other members, such as how to communicate with friends and relatives regarding their illness.

“When I see what others are doing, I can push myself harder.” (Participant 5)

Group therapy had a range of other benefits, such as participants being more likely to discuss and share the experiences on: medication use and side effects; treatment in the outpatient and inpatient unit; experience with family members and friends; and varying coping strategies that they found useful.

However, a few participants suggested that individual therapy sessions would have been better because they experienced difficulties talking in a group with 8–10 people. Some participants felt embarrassed after sharing their own experiences about the illness with others, because someone in the group was laughing.

“I was sharing my own experiences at the period of manic onset; I told everyone I earned salary of 10000 RMB in a month, my husband only earned 8000 RMB. I wanted to change my husband at that time….but then everyone in the group was laughing at me and I felt very embarrassed.”

(Participant 13)

Two participates reported that they felt uncomfortable at first when discussing each other’s problems in front of strangers.

“I didn’t know what to expect from the course and I felt very anxious initially…many strangers were siting around me.” (Participant 3)

#### Sub-theme 2: Class-taught versus discussion

Most of the participants preferred to be involved in the facilitator-directed class discussion compared with a teacher-directed class. Their reasons included difficulty maintaining attention when all they could hear was the teacher talking. An effective way to improve interest may be to require participants to think and connect with the content.

“If I only listened to the teaching talking, I would lose my interest in the class.” (Participant 5)

A few participants reported that they preferred teacher-directed classes, because some participants from the group were likely to dominate the group discussion.

“She kept talking, talking and talking in the class, I felt restless and annoyed at her.”

(Participant 2)

#### Sub-theme 3: Family involvement

Altogether, 80% of the participants mentioned that they would like to ask their family members to become involved in the program. Listening to the class would help some family members understand that they should not put too much pressure on BD sufferers, because some of the symptoms are due to biological or environment effects, rather than “stubbornness”.

“If my mom could come to the class that would be helpful, she could listen more about bipolar disorder, the medication I am taking and this would help her understand more about me….maybe she will change her attitude towards me, that I am not lazy.” (Participant4)

Some participants suggested that it was only necessary for their family to participate in some sessions, because some of the sessions are not relevant to them.

“Yes, especially in the psychological session, the doctor taught us how to identify, understand, express and manage our emotion; if my husband was here, it would definitely improve our relationship.”

(Participant 13)

However, some participants did not show this interest; one participant expressed that their family members lived far from the hospital and would have trouble with transportation and taking time off work.

“That’s too far, my dad is living in the Shandong province, I don’t want him to come here everyday, you know traveling is difficult for him”.

(Participate 14)

Two other participants reported that they had poor relationships with their mom/husband and would prefer not having them within the group.

“No, I don’t. I don’t want my mom to participate in the class because we argue very often at home”. (Participant 2)

#### Sub-theme 4: Real stories sharing

Some participants suggested that the course could include one or more patients with bipolar disorder who had been cured or who are effectively managing their illness. Hearing from such individuals could inspire hope and confidence that recovery is possible for others.

“It’s better to listen to some real stories from real people who have been cured, such as how they recovered from the bipolar disorder, what they did during the treatment and recovery period… which medication they think is the best….this information could help me feel more authentic”. (Participant 15)

### Theme 4: Perception of learning content

Most of the participants commented that the content was professional, well structured and useful. The following results are specific to each session.

#### Sub-theme 1: Concept of BD

Participants said that the session on “bipolar disorder” increased their knowledge of BD, such as the symptoms of depression, manic behavior, and etiology.

“No one has ever told me what is bipolar disorder, and now I know what has happened to me.” (Participant 10)

#### Sub-theme 2: Treatment of BD

Most of the participants expressed that they were willing to take different medications for Bipolar disorder, and that they understood how important the medications were as an adjunct to other psychotherapeutic intervention.

“I don’t like to take medication, I always hide the medication under my pillow and then the nurse found out and forced me to take it. But now I understand why I should take the medication.” (Participant 13)

#### Sub-theme 3: Psychological approach

Some patients participated in psychological exercises, such as imagery relaxation and group-based mindfulness practice. These were experienced as being useful and patients kept practicing them in their daily life. This session also included some basic cognitive therapy.

“The imagery relaxation made me feel comfortable; I could imagine that I was in the ocean with my lover. I also practiced it before going to sleep every night and it enabled me to really calm down.” (Participant 9)

Some participants expressed that they had learned how to replace some automatic negative thoughts and beliefs with new rational beliefs, which lead to their behavior becoming more reasonable. Particularly, two participants mentioned that they would continue to participate in cognitive behavioral therapies after their discharge from hospital.

“In the past, I was always thinking that I was going to die, but I understand now this type of thought is called catastrophizing; catastrophizing means only expecting the worst outcome in everything, so actually I was not going to die.” (Participant 7)

#### Sub-theme 4: Self-management

Some participants reported that they found the self-management session useful, such as learning how to list triggers and monitor mood. Participants’ emotions have become more stable as a result of the self-management session; they know how to recognise, understand, express and self-manage their emotions.

“I feel that I can actually recognize the dangerous triggers in the different environment settings as a result of an increased awareness of my mood.” (Participant 5)

#### Sub-theme 5: Lifestyle management

All participants reported that maintaining a regular bio-clock rhythm, being physically active, and abstaining from alcohol, reinforce the importance of a healthy diet and lifestyle.

“I always reversed my bio-clock, I used to work at night until 5 to 6 am and go to bed at daytime. I understand if I do not want to come back to hospital again, I should have a regular life.” (Participant 7)

“I will keep doing sports every day because my mood becomes more stable after playing badminton with my neighbor every time.” (Participant 6)

#### Sub-theme 6: Relapse prevention

Some participants reported that the mood diary was the most helpful tool. They reported that monitoring their mood changes on a day-to-day basis would assist in making the most of their visit to see the doctors.

“The mood diary was the most useful thing I have learned from the class, it can help me monitor my mood, if the score is higher than 4 or 4.5, I should definitely see my doctor as soon as possible.”(Participant 8)

#### Sub-theme 7: Suggestion for improving the learning content

Some participants suggested ways in which the content could be improved. Participants complained that they still had fears of modified electroconvulsive therapy (MECT), and therefore they thought the program should include more details on MECT, such as its mechanism of operation, side effects, and information on anesthesia.

“I am still not very sure of electroconvulsive therapy, what’s that for? Will I die after injecting something in my body by the doctor?” (Participant 8)

Participants also thought that the mood charting exercise should contain more examples of how to complete the diary.

“The doctor gave me the sheet but I totally had no idea how to do that.”(Participant 13)

Participants also suggested wanting to understand how to reduce or manage the side effects of their medication.

“The side effects of the medication are common to everyone, not only me, but I still fear weight gain as result of taking long-term medication, I want to know how to reduce this side effect.” (Participant 8)

“I understand how important medication is to me, but the side effects….You know I am a girl and I am not married yet.” (Participant 7)

Some female participants suggested that the content should include information about bipolar disorder and pregnancy.

“I have bipolar disorder and want to get pregnant but I want to know the risks and benefits of medications and forms of birth control.” (Participant 13)

### Theme 5: Outcome of participating in program

#### Sub-theme 1: Self-acceptance

The majority of participants reported that the program reduced their feelings of embarrassment, shame and confusion regarding their illness. Hence, the program increased their acceptance of their illness.

“I understand that my illness is not my fault.” (Participant 11)

“This is something I can’t change by myself, but I should accept it.” (Participant 15)

#### Sub-theme 2: Self-confidence

Some participants mentioned that they were struggling to speak up for themselves within their daily lives. However, whilst practicing skills introduced through group work, patients increased their confidence for confidently interacting with people outside the group.

“I was struggling to speak up about my illness, but when someone was speaking up about their illness in the group discussions, that would give me the confidence to do the same.” (Participant 8)

“Since I started to share my own experiences in the group, I felt stronger within myself.” (Participant 6)

#### Sub-theme 3: Self-awareness

Some participants reported that they had become more aware of warning signs of a bipolar episode, and this helped them address their mood when it was becoming excessively high or low.

“Once I recognized the trigger and in the future I will know when my mood is becoming low or high.” (Participate 15)

#### Sub-theme 4: Self-motivation

Some participants stated that they experienced increased motivation to participate in treatment and engage in lifestyle changes, and these feelings led to a sense of control.

“My mom sent me to the hospital because I would always go on a shopping sprees... If I receive enough treatment, I won’t waste the money again.” (Participant 4)

“I feel so sorry and regret towards my husband, I won’t try to change my husband again despite my salary being higher than him, I will try my best to recover from the illness.” (Participant 13)

#### Sub-theme 5: Social support

Some participants reported that they developed new friendships with group members and that they would continue to meet each other and offer social support at the end of the program.

“I am not only getting support from my family, but also from the group members.” (Participant 8)

“She said I am her good friend, we did everything together during the hospitalization, we would wake up at the same time, go to the washroom, watch TV, sit together when eating breakfast and lunch.” (Participant 6)

#### Sub-theme 6: Relationship with the doctor

Some participants mentioned that the program enhanced the relationship with their doctor and they also reported an increase in trusting their resident doctor at the end of program.

“I was reluctant to talk to my resident doctor before attending this program, because I thought he had lied to me about my condition and that he just wouldn’t let me go home but I found out I was wrong….we did have a pleasant conversation yesterday in the ward and I believed him.” (Participant 12)

Some participants said this change in trust may result in greater adherence to outpatient visits and check-up sessions.

“If I keep up taking the medication that my doctor prescribes me then everything will be okay for me.” (Participant 5)

## Discussion

### Perception of participating in the program

Those patients who showed a higher understanding of their illness would encourage positive treatment outcomes, and this formed the main motivation to complete the program. The participants showed interest in recommending the program to others. These findings are in agreement with an earlier qualitative study in outpatient sample [8]. The patients recommended that the program should provide a comprehensive educational booklet. This may be due to some patients favouring written materials as they can then review them at any time [21]. Finally, participants expressed interest in further participating in follow-up studies after discharge from the hospital.

### Perception of the setting

Most of the participants reported that the learning environment facilitated their attention and attendance, with the presence of light music enhancing their mood and focus while in class. The single-table seating led to feelings of equality and solidarity. Some participants suggested that the learning environment could improve, with suggestions including enhancing the ambient light and introducing breaks and refreshment, as well as ensuring a quiet and undisturbed environment.

Participants reported preferring afternoon sessions due to morning sleepiness and the disruption to preferred daily routines. Instead of attending classes in the mornings, participants would like to attend classes during the afternoon, perhaps after a nap. Most participants agreed that each session should take about 40 min to achieve a balance between the content covered and the toll on attentional and emotional resources. All participants in our sample were presently in a recovery period from acute BD symptoms and therefore they could experience disruptions to mood during longer sessions. The present study agreed that each session should have intervals separated by 2 to 3 days as participants may lose interest and patience to the program if the gap between sessions is excessive. Our program delivered eight sessions in a period of two weeks. This might explain why the drop out rate was low in our study.

Most of the facilitators were perceived as professional, friendly, respectful and responsible. There were suggestions for the facilitators to: (i) improve the powerpoint slides to include more interesting, flashy information, photos and videos, in order to enhance the class’s learning atmosphere; (ii) expand even further upon main points offered in the lecture slides; (iii) allow more time to answer participants’ questions; (iv) improve class-management skills to prevent dominant group-members offering disruptive or irrelevant content. Indeed, in a study by Poole, Smith and Simpson [8], it was found that participants dominating the group members was one of the reasons for dropping out the program. Lastly, participants suggested that the facilitators (v) should offer a revision of the previous session at the beginning of each new session, as participants reported difficulties remembering all the information.

### Perception of the learning content

The content and the lecturer were both judged of being of expert quality, clear and well structured. Participants especially enjoyed the sessions that included: definition of bipolar disorder; a treatment approach; a psychosocial approach; self-management; and healthy lifestyle in order to prevent relapse. However, the lack of detailed knowledge about MECT caused some participants to feel anxious. Hence, participants suggested that the MECT session should cover the mechanisms of operation, side effects, anxious relaxation and some basic knowledge of anesthesia. More information about bipolar disorder and pregnancy and how to reduce the side effects from the medication were requested from female participants. Female patients appeared more prone to worry about their weight gain as result of taking medication. Excessive weight gain was the most common cause of non-adherence of treatment [22]. Crucially, further instruction and practice on mood charting is necessary as most of the participants reported that they did not know how to complete the exercise sheet at the end of program.

### Perception of participating in a group

Most of the participants stated that the group-based psychoeducation was preferred over individual intervention. This result supports earlier findings, as learning that the patient is not alone in suffering the illness can alleviate feelings of shame and isolation [23]. Participants were able to learn how to communicate with their friends and relatives from interacting with the group. In the Chinese society, the role of “teacher” has long been respected. Chinese people think the teacher is the expert they can trust and rely to receive valuable suggestions [24]. Interestingly, there are psychotherapy studies, such as those including cognitive behavior therapy, suggesting that didactic teaching is the desirable approach during the treatment process, since it can improve the trust relationship between patients and psychotherapists [24–26]. Surprisingly, in our study, most of the participants preferred to be involved in a facilitator-directed class discussion rather than in didactic teaching. Some participants felt embarrassed after sharing their own experiences as some group members were laughing at them. In order to partially address these concerns, perhaps the facilitator should remind participants of the principle of information confidentiality. Also, the facilitator should emphasize the key points from the slides by asking questions to participants, hence refocusing any discussion that approaches an irrelevant topic. For those participants who feel uncomfortable during initial sessions, the facilitator should provide a more natural icebreaker exercise [8]. Many participants suggested that involving family members would be useful, as this would promote an understanding of the patient’s illness and their current condition. However, whether the relatives should become involved in the program depends on the patients’ personal and financial situation. Poole, Smith and Simpson [8] suggested that future programs could provide sessions specifically for family member to become involved in the therapeutic process. Lastly, some participants suggested that the program should invite patients who are effectively managing or have been cured of BD. Sharing their treatment and recovery experiences with the group may encourage hope and confidence within these group-members. This point also was raised in previous study [8].

### Outcome of participating in the program

Poole, Smith and Simpson [8] demonstrated that a UK-based group pychoeducation intervention had positive impact on the patient’s knowledge, acceptance, social support and attitude towards taking medication. Our results are comparable to findings from European patients, as participants reported that the program increased their personal acceptance of their illness, reducing their feeling of confusion and self-doubt. Patients became more confident to communicate with the people outside the group, which can reduce self-stigma and promote prosocial behaviours. A number of participants stated that they have a strong motivation to stay in treatment and experience lasting changes as a result. Some participants reported that they would continue to meet each other at the end of the program, therefore increasing their social support. Some participants increased their awareness as they became more capable to recognise personal triggers. Others reported that the program enhanced their “trust relationship” with their doctor. A more trusting relationship could increase compliance and help achieve better treatment outcomes [27].

### Strengths and limitations

The aim of this study was to qualitatively explore experiences of group-based psychoeducation for BD-I via thematic analysis. The data analysed during the process evaluation helped the authors understand the strengths and weaknesses of the program. This addresses a gap within existing literature regarding the efficacy of group psychoeducation for individuals with BD. This study is the first to assess group-based psychoeducation programs for inpatients following remission of a manic episode in bipolar I disorder within a Chinese sample. The findings indicate that the program was helpful for patients with BD-I. Patients offered a range of recommendations that future research on group-based psychoeducation programs should explore. There are some limitations in this study: the methodological design did not include direct questions asking for participants’ potential discomfort and non-beneficial aspect of the program; our sample had a large proportion of female patients, hence future studies may wish to explore differences in group dynamics across a range of gender ratios; family was not involved in this study, so future research needs to explore family-based intervention for BD in Chinese populations; finally, since this study was conducted on a single hospital ward, future research may consider implementing this intervention across multiple sites and include community based psychoeducation programs to explore its generalisability.

### Clinical implications

Future programs should include further details about MECT and more information about BD and pregnancy for female patients. More support from facilitators is required when patients work on mood charting. Importantly, participants may benefit from less didactic teaching and more discussions during each session. Finally, it would be helpful for patients to have access to a comprehensive education booklet, which they can then review at any time, at their own pace.

As recommended by many participants, future psychoeducation education programs should involve family members, as this could help them better understand the patients’ illness and current condition. Future programs may also consider to invite BD patients who have been successfully treated, as sharing their treatment and recovery experiences with the group may encourage hope and confidence.

Our study demonstrated that a culturally adapted group-based psychoeducation program for BD is feasible and acceptable in a Chinese population. Different social and cultural backgrounds could impact on the patient’s perception of their symptoms and services engagement [28]. The role of Confucianism in the Chinese culture as well as its collectivistic tradition discourages open displays of emotions in order to maintain social and familial harmony or avoid exposing personal’s weakness/perception. As a result, some Chinese patients are reluctant to share negative feelings due to fears of creating interpersonal conflicts (and hence damage their connections with others) and bring shame to their family (“lose face”), which could result in the avoidance of interactions in group interventions. For instance, Lin [25] indicated that Chinese American patients may experience difficulties with straightforward discussions about some problems between family members during cognitive behavior therapy. Thus, when working with Chinese patients, the notion of “saving face” is an important element that needs to be considered through the course of intervention [24]. In our study, we found that most of the participants were willing to discuss and share personal experiences with others in the group. Facilitators can therefore educate patients to recognize the importance of actively engaging in group discussions in the beginning and during the course of the sessions. Also, culturally adapted interventions are an effective way to facilitate group discussions and promote a good trusting relationship, such as using local folk stories, idioms, image and examples from religious [28].

## Conclusion

This qualitative study provides positive evidence on the benefits of a group-based psychoeducation program in enhancing patient knowledge, confidence, acceptance, motivation, social support and a trusting relationship with the doctor. Based on the benefits of our program on patients, this intervention could potentially be incorporated into routine psychiatry inpatient care. Therefore, it is suggested that policy makers should consider placing more efforts on the provision of more accessible psychoeducation interventions to patients with bipolar disorder in China.

## Abbreviations

BD: Bipolar disorder
BD-I: Bipolar disorder type I
